# Perceptions of the PrePex Device Among Men Who Received or Refused PrePex Circumcision and People Accompanying Them

**DOI:** 10.1097/QAI.0000000000000703

**Published:** 2016-05-24

**Authors:** Minja Milovanovic, Noah Taruberekera, Neil Martinson, Limakatso Lebina

**Affiliations:** * Perinatal HIV Research Unit, Faculty of Health Sciences, University of the Witwatersrand, Johannesburg, South Africa;; † Population Services International, Johannesburg, South Africa; and; ‡ Johns Hopkins University Centre for Tuberculosis Research, Baltimore, MD.

**Keywords:** attitudes, MMC, device circumcision, South Africa

## Abstract

**Background:**

The PrePex medical male circumcision (MMC) device has been approved for MMC scale-up. However, the WHO has recommended that a country-specific situation analysis should be carried out before MMC device rollout.

**Method:**

A cross-sectional survey was conducted over 12 months in 3 MMC clinics, by trained nurses and researchers, to ascertain attitudes toward PrePex MMC in 3 groups: men consenting for PrePex MMC (PrePex recipients), people accompanying men, and adolescents coming for either PrePex or surgical circumcision (MMC escorts) and men refusing the PrePex device MMC (PrePex rejecters). All participants received information on surgical and the PrePex device MMC methods.

**Results:**

A total of 312 PrePex recipients, 117 MMC escorts, and 21 PrePex rejecters were recruited into the study. Ninety-nine percent of PrePex recipients thought that their expectations (safe, convenient, minimal pain) were met, and they were pleased with cosmetic outcome. Fifty-nine percent of PrePex rejecters opted for surgical circumcision because they perceived PrePex to be novel and risky. All 3 groups of participants were concerned about odor, dead skin, discomfort, healing time, and wound care. Ninety-eight percent of MMC escorts, 99% of PrePex recipient, and 81% of PrePex rejecters perceived PrePex circumcision as an acceptable option for South African MMC programmes.

**Conclusions:**

This acceptability study suggests that PrePex MMC is considered safe and convenient and could be incorporated into existing MMC programmes. Concerns about odor, pain, wound care, and healing time suggest that the need for more research to further optimize methods and that MMC clients should be counseled on available methods to enable them to choose among options based on their preferences.

## BACKGROUND

The PrePex medical male circumcision (MMC) device was prequalified by the World Health Organisation (WHO) in May 2013.^1^ The WHO has recommended that before broad programme implementation of MMC devices, country-specific implementation pilot studies be performed to identify any cultural sensitivities, gender issues, and religious beliefs around device MMC.^2^ If context is not considered, programme implementation of this innovative method may be jeopardized.^2^ PrePex and the Shang Ring MMC devices have been evaluated for safety, acceptability, and efficacy in Africa.^3,4^

South Africa has the highest absolute number of people living with HIV globally, and in the 2012 population, HIV prevalence was 12.2%.^5^ Mathematical modeling studies have indicated that circumcising 4.3 million by 2016 would avert 1 million new HIV infections.^6^ However, South Africa is far from its target of circumcising 80% of males aged 15–49 years.^3,7^ For the period 2013–2014, a reported 512,902 MMCs were performed in South Africa.^8^ Barriers previously identified for the uptake of MMC include medical costs, opportunity costs such as lost income due to time away from work, fear of pain, concern about safety due to surgery, adverse events, and postprocedure sexual abstinence.^9^ New methods of circumcision should address acceptability of MMC to promote demand and minimize any barriers to uptake.

Although PrePex has been marketed as a quicker and safer MMC option that requires no local anesthesia injection, no cutting of live tissue, and no suturing, there is limited information on how PrePex may be perceived and accepted by stakeholders in South Africa. South Africans may be particularly culturally sensitive about circumcision, as it is linked to rites of passage into manhood in some groups.^10^ Therefore, new methods of MMC should be evaluated for cultural appropriateness, perceptions, and acceptability by potential users and those close to them before implementation. We assessed cultural appropriateness, individual perceptions, and acceptability of PrePex in South Africa.

## METHODS

A cross-sectional survey of attitudes toward PrePex MMC was conducted between August 2013 and July 2014 in 2 high-volume MMC clinics (Witbank Hospital and Tsakane Clinic) and 1 HIV wellness and MMC clinic in Johannesburg (ZuziMpilo clinic). We approached 3 groups of participants: men consenting for PrePex MMC (PrePex recipients), people accompanying men and adolescents coming for either PrePex or surgical circumcision (MMC escorts), and men refusing the PrePex device MMC (PrePex rejecters). Study personnel (trained nurses and researchers) interviewed participants postcounseling but before circumcision procedure. Men who received PrePex MMC were also interviewed during their termination follow-up visit at day 56 postplacement. All eligible participants who were approached consented to being part of the study.

### Settings

The Witbank and Tsakane sites were established by the Society for Family Health (SFH), a South African affiliate of Population Services International, with funding from the Centre for Disease Control (CDC) South Africa. They are massive Voluntary Medical Male Circumcision sites that provide free HIV testing and counseling and MMC services for adults and adolescents. Any male above the age of 15 years is eligible for MMC at the 2 sites. Each site performs an average of 800–1200 MMCs per month during winter (May–August), while in low-demand periods the sites perform approximately 250–400 MMCs per month.

ZuziMpilo, an HIV wellness and MMC clinic, is situated in downtown Johannesburg and is accessed mainly by people without medical insurance. The primary services offered are chronic disease management especially for HIV. Over 1200 MMCs had been performed between 2010 to the time we recruited patients to this study.

### Study Procedure

The 3 sites used similar study procedures and data collection tools. Participants were recruited from the study sites based on whether they consented to have free-of-charge circumcision with the PrePex device, were accompanying a client coming for MMC, or refused the PrePex device MMC. Participants in the PrePex acceptor and PrePex rejectors groups received information during the counseling sessions on both forceps-guided surgery and the PrePex device MMC to be enabled to choose the method best suited for their individual needs. They were then individually consented into the study if they were willing to participate. Participants from all 3 groups had to be above the age of 17 years to be eligible for the acceptability interview. Men consenting to PrePex were the only group who were interviewed twice: before having the PrePex procedure and at their termination follow-up visit. People accompanying men and adolescents coming for MMC attended the same counseling as the MMC clients and were told about the PrePex device and procedure and surgical circumcision. All men refusing PrePex opted for forceps-guided surgical circumcision instead.

### Data Collection and Analysis

A structured questionnaire with open-ended and closed-ended questions was administered to explore perceptions and acceptability of the PrePex device.

Comments and views of study participants were collected and are presented as a means to demonstrate attitudes and perceptions of PrePex. The 6 steps of thematic analysis given by Braun and Clarke^11^ were used to analyze the qualitative findings. This method allows for the categorization of patterns of data and insight into individual and collective perceptions of the PrePex device MMC. Quantitative data were analyzed descriptively using SPSS version 22 (IBM Corp. Armonk, NY) and were used to support qualitative findings. The qualitative quotes are presented in their original format.

### Ethics Statement

The study was approved by the Institutional Review Board of the University of the Witwatersrand Ethics Committee.

## RESULTS

A total of 312 PrePex recipients, 117 MMC escorts, and 21 PrePex rejecters were recruited into the study. Of the 312 PrePex recipients, 257 (82%) answered the questionnaire at their last follow-up visit. The median age for PrePex recipients was 26 years [interquartile range (IQR): 22–30 years], for MMC escorts was 36.5 years (IQR: 30–45 years), and 29 years (IQR: 24–33 years) for PrePex rejecters. The leading language spoken by all 3 groups was Zulu. The majority of MMC escorts were family members (partners, parents, and siblings). Both PrePex recipients (40%) and PrePex rejecters (55%) said that someone else influenced their decision to go for MMC (*P* = 0.17). Although all participants consented to the questionnaire, they did not answer every question. The median item response (qualitative and quantitative) across the 4 questionnaires was high, with 98% (IQR: 97%–99%) of all items answered by PrePex recipients during placement interview, 96% (IQR: 91%–98%) during last follow-up interview, 95% (IQR: 91%–96%) of all items were answered during MMC escorts interview, and 95% (IQR: 90%–100%) of all items were answered by PrePex rejecters.

### PrePex Recipients

#### PrePex Recipients' Perceptions Before MMC

The PrePex recipients described circumcision as a safe, clean, and protecting from diseases and that it will make them socially acceptable. The reasons they gave for choosing medical over traditional circumcision include closer to home, free, done by professionals, and not seasonal.

For many PrePex recipients, the decision to use PrePex was based on being afraid of blades, injections and stitches, ability to return quickly to daily activities and perceptions that the PrePex MMC wound would require less care. “*Because it is safe comfortable and infection are minimal there is no way of losing your penis*” (T095, 21 years). A large majority (87%) of PrePex recipients also thought that PrePex MMC was safer than blade-based circumcision (surgical and traditional) and 99% stated that they trusted PrePex (Table 1).

**TABLE 1.**
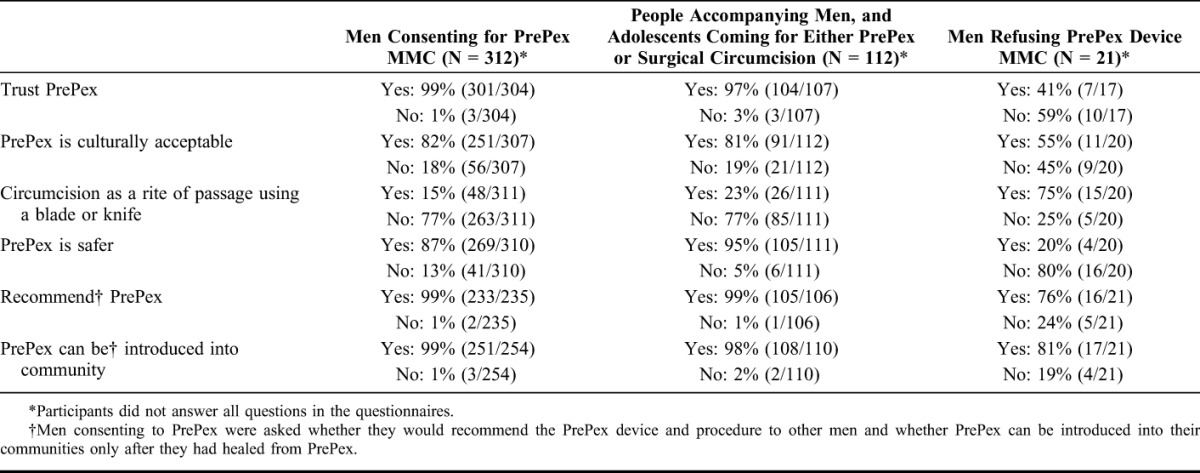
Perceptions of PrePex Across the 3 Participant Groups—Quantitative Findings

#### PrePex Recipients' Perceptions After MMC

The experience of PrePex recipients once the MMC wound had healed was that the procedure was good, hygienic, safe, and convenient. Some said it had minimal pain, and they were comfortable enough to go to work and walk around, “…*was expecting to experience more pain and discomfort only to find that pain was bearable*…” (T037, 23 years). Almost all (99%) PrePex recipients were also pleased with the cosmetic outcome at the last follow-up visit.

Features that PrePex recipients did not like about the PrePex procedure included discomfort while wearing the device especially when having an erection; the dead, foul smelling, black foreskin; difficulty passing urine; and oozing. They also found that it was not easy to bathe with device and that the wet sloughy wound after removal adhered to underwear “*The device was too tight and it was painful. After removal the penis likes to stick on his underwear and the painful erection and washing it after removal the salt was so uncomfortable*” (Z035, 37 years). Delayed healing and late resumption of sexual activity were also issues. Some of the participants were tempted to remove the PrePex device early because of the blackening foreskin, pain, and discomfort, “*I almost removed it as I was scared especially when the skin turned black*” (T019, 23 years).

When the participants were questioned if PrePex met their expectations, 97% (247/255) said “Yes” because the explanations they received had prepared them for the procedure. Although some were anxious before the procedure and expected complications because PrePex is a novel method, they were satisfied both with the procedure and the outcome, “*Yes at first I was stressed asking myself if I did the right thing but now I see I chose the good method*” (Z082, 32 years). When directly asked, a few (5.4%; 14/258) participants complained that the procedure was more painful than the explanations before the procedure had led them to believe. Furthermore, most participants said the procedure was fast although a few said it took long.

When the PrePex recipients were asked what was most challenging with PrePex, they cited pain and odor at the time of removal, painful erections and difficulty sleeping, disturbances in urine flow, keeping the wound clean after removal, and waiting until complete healing before resuming sexual activities. “*It was a roller coaster, one day you feel you did the right thing but the next day you feel you did the wrong thing*” (Z047, 36 years).

Virtually, all (99%) PrePex recipients, at last follow-up visit, thought that the PrePex device and procedure could be introduced into their communities, and they would also recommend it to other men. However, when specifically asked if they would choose the PrePex device again 5% said no because PrePex takes long to heal, odor, and surgical MMC is better known, and more men have done it.

### MMC Escorts' Perceptions

This group considered any MMC to be hygienic, safe and a way of minimizing injuries during sex and preventing diseases. They also thought men who are circumcised are more socially accepted by their peers as they would be regarded as mature “*Be born again as a man*”(W504, 21 years).

Following an information discussion on PrePex, MMC escorts felt that some of the perceived benefits of PrePex were it is safe (placed outside of the body with no cutting), faster, clean, and could be easily accepted by those scared of “sharps.” For some, the PrePex method reminded them of other medically accepted procedures, “*Similar to cutting umbilical cord of baby*” (Z502, 54 years). MMC escorts thought that wearing the device for 7 days and the smell of the dead skin might make it uncomfortable and undesirable for some, “*Looking at your penis and it's rotten and smelling*” (Z510, 20 years). Some of the MMC escorts concerns included difficulty with bathing and the device falling off before scheduled removal.

Eighty-one percent of MMC escorts thought that the PrePex device was culturally acceptable because traditional circumcision is more concerned with teaching values than the method used for foreskin removal. Minimal pain was considered as an advantage by many; however, a few thought that this feature was not culturally acceptable because it would not teach a boy to be a strong man.

Almost all MMC escorts (98%) believe that PrePex can be introduced into their communities and 99% said that they would recommend it to others. “*There will be less uncircumcised men in South Africa because most men are afraid of bleeding*” (W500, age not disclosed).

### PrePex Rejecters' Perceptions

In general, PrePex rejecters considered circumcision as an important culturally acceptable and healthy way of protecting themselves from diseases. Although 75% of PrePex rejecters agreed that traditional method of circumcision uses a blade or knife, they did not think that culture influenced the method of circumcision—as long as the foreskin had been properly removed.

PrePex rejecters thought PrePex was a good alternative to surgical MMC because it has less pain, looks safer, requires no injection, and is suitable for those who are scared of surgery, “*Because some people are scared of surgical they will prefer PrePex*” (W316, 24 years). However, they felt that appropriate information has to be provided and the man must have time to return to the clinic for follow-up visits. PrePex rejecters chose not to have PrePex because it is a new device in South Africa, and it was considered risky, with 80% (16/20) of PrePex rejecters judging it as less safe than surgical MMC. Furthermore, factors that influenced the PrePex rejecters decision were not wanting to be part of a study, lengthy procedure time, and prolonged time to healing compared with surgical, “*Because its still a study and its new and more risky*” (W302, 31 years). Based on these reasons, more than half (59%) of PrePex rejecters said that they do not trust the PrePex device (Table 1). Furthermore, they perceived the use of a plastic ring that stays on for a week as a feminine act, “*No, a man should not put stuff underneath like woman*” (W302, 31 years). Despite this, many PrePex rejecters (76%) felt that they would consider recommending PrePex to other men and the majority (81%) agreed that it could be introduced into South African MMC programmes.

## DISCUSSION

Our study suggests that the PrePex device MMC is considered to be acceptable, safe, and easy by PrePex recipients, MMC escorts, and PrePex rejecters and could be introduced into South Africa. At the same time, PrePex recipients identified problems with PrePex such as pain, odor, and issues relating to healing. Prior reports suggest that MMC for HIV prevention is not accepted by all men^12^ because of pain, sexual abstinence, fear of complications, loss of income, and religious reasons.^13^ However, those who select PrePex are often satisfied with the outcome.^14^

Device-based MMC procedures may be preferred over blade-based circumcision (surgical or traditional), and a previous study showed that 82% of men selected the ShangRing over surgical MMC.^14^ Some of the perceived benefits of device-based MMC as reported by participants in this and other studies are ease and rapidity of application and minimal disruption of daily activities.^4,15,16^

An important finding across studies is that majority of participants are satisfied with the cosmetic appearance resulting from both ShangRing and the PrePex device MMC and therefore would recommend it to their friends and family.^14,17,18^ Participants in other studies have also raised concern regarding odor while wearing the PrePex device,^16^ pain during removal, and need for a follow-up visit for removal.^4,15,19^ Therefore, clear and appropriate messaging on advantages and disadvantages of the PrePex MMC should be given to avoid misunderstandings about the procedure.^9,16^

There are no published studies we are aware of that report the acceptability of device-based MMC among partners, friends, and family members, and those who refuse to have a device-based circumcision. Our study found this method to be culturally appropriate and an acceptable alternative to surgical circumcision. However, most respondents were concerned that PrePex is novel and that more research from within South Africa would be reassuring.

## CONCLUSIONS

This acceptability study, from 3 sites, in South Africa suggests that PrePex MMC is considered acceptable by men consenting for PrePex MMC, people accompanying men, and adolescents coming for either PrePex or surgical circumcision, and men refusing the PrePex device MMC and that it could be implemented into existing MMC programmes. However, concerns about odor, pain, wound care after removal, the lengthy healing, and sexual abstinence periods suggest that potential MMC clients should be counseled on all available methods to enable them to choose the one best suited for their needs. There is also a need for more research to minimize some of the observed challenges with PrePex MMC.
